# Refractory Myasthenia Gravis and Concurrent Alopecia Areata Postthymectomy With Improvements After Cortisone Taper: A Case Report

**DOI:** 10.1155/2024/5556012

**Published:** 2024-09-19

**Authors:** Baraa Alghalyini, Huda Dahman, Abdul Rehman Zia Zaidi, Fathima Aasiya Tehreemah Raziq, Mohammad Amin Alswes

**Affiliations:** ^1^ Department of Family and Community Medicine College of Medicine Alfaisal University, Riyadh, Saudi Arabia; ^2^ College of Medicine Alfaisal University, Riyadh, Saudi Arabia; ^3^ Department of Family Medicine King Faisal Specialist Hospital and Research Center, Riyadh, Saudi Arabia

**Keywords:** alopecia areata, cortisone taper, postthymectomy, refractory myasthenia gravis

## Abstract

This case report presents a unique clinical scenario of a 58-year-old male suffering from severe refractory myasthenia gravis and concurrent alopecia areata postthymectomy. Myasthenia gravis, a common autoimmune disorder, is characterized by muscle weakness due to autoantibodies targeting neuromuscular junction proteins. Alopecia areata, another autoimmune disease, is often seen in individuals with myasthenia gravis, suggesting a shared immunological basis. The patient's condition was resistant to conventional treatment, and he developed alopecia areata following thymectomy. Despite the challenges in managing refractory myasthenia gravis and the associated alopecia areata, significant improvements were observed following a cortisone taper. This case highlights the potential therapeutic role of cortisone tapering in managing refractory myasthenia gravis and associated alopecia areata. This case also prompts further exploration into the immunological shifts following thymectomy, particularly its potential role in triggering alopecia areata.

## 1. Introduction

Myasthenia gravis is one of the most frequent conditions affecting the neuromuscular junction (NMJ) and is an organ-specific autoimmune disorder that causes weakness in skeletal muscles. The muscle weakness results from the diminished electrical impulse transmission across the NMJ caused by the development of autoantibodies against the postsynaptic membrane proteins. The main symptoms of myasthenia gravis include ptosis (sagging of the eyelid), diplopia (double vision), dysphagia (difficulty swallowing), dysarthria (difficulty speaking), and muscular weakness in extremities and the neck [1, 2]. A major complication of myasthenia gravis is the myasthenic crisis which is an acute respiratory paralysis [1]. Myasthenia gravis is common in both males and females of all ethnic backgrounds. Although it can happen at any age, including childhood, it most frequently affects young adult females (under 40) and older males (over 60).

Refractory myasthenia gravis is classified as a poor response to conventional treatment or recurrent myasthenic crises despite immunosuppressive and symptoms-based therapy [3]. Risk factors for refractory myasthenia gravis include being female with an early age of onset, as well as the presence of anti-MuSK antibodies and thymomas [4].

Myasthenia gravis is a well-known paraneoplastic disease of thymoma, with up to 50% of thymoma patients experiencing myasthenia gravis. Thymomas are uncommon tumors that develop in the anterior mediastinum from the thymus [5]. Thymus is thought to contribute to the development of myasthenia gravis as it is considered the primary site for the aberrant synthesis of the antibodies implicated in myasthenia gravis. It was determined that thymectomy (removal of the thymus gland) improved prognosis, particularly in young patients when carried out as soon as feasible. This is because the risk of acetylcholine receptors (AChR)-specific T lymphocytes leaving the thymus and migrating to the lymph nodes and peripheral lymphoid tissue can be reduced by total thymectomy and is indicated in all patients with thymoma associated myasthenia gravis [6–9].

Thymectomy, while primarily therapeutic, may also disrupt immune homeostasis, potentially triggering or exacerbating other autoimmune conditions such as alopecia areata. This complex interplay highlights the need for careful postoperative monitoring and consideration of emerging or worsening autoimmune symptoms.

One organ-specific autoimmune phenomenon that is associated with myasthenia gravis is alopecia areata, which is marked by a sudden onset of nonscarring hair loss and bald patches that are well-circumscribed and oval or circular in shape [10, 11]. Myasthenia gravis and alopecia areata represent primary pathogenic pathways involving two distinct adaptive immune system arms; myasthenia gravis is B cell-mediated, while alopecia areata is characterized by damage of the hair follicle due to infiltration of the cytotoxic subset of CD8+ and NKG2D+ T cells into hair follicles [12–14]. As it is common for these two conditions to occur together, it is thought that they may share immunological foundations. Myasthenia gravis is frequently associated with thymoma, and there have been several reported cases of alopecia areata linked to either thymoma and/or myasthenia gravis [12]. More specifically, although alopecia areata concurrent with thymoma has been noted to be common in literature, alopecia areata associated with myasthenia gravis alone has been noted to be rare [15]. However, there have been cases such as those reported by Kubota, Komiyama, and Hasegawa [16] and Suzuki, Utsugisawa, and Suzuki [17], where a small percentage of patients with myasthenia gravis were discovered to have both alopecia areata and thymoma. The study by Suzuki et al. (2005) in particular identified a subset of patients (6 out of a cohort of 159) with myasthenia gravis who developed alopecia areata after thymectomy, much like our own patient. Their data suggested that these patients are more susceptible to other autoimmune diseases such as myositis, myocarditis, and Hashimoto's thyroiditis, especially if they have had a severe neuromuscular presentation and an extended period postthymectomy. Thymectomy affects the phenotype of peripheral blood T cells, and hence, this change may lead to the development of alopecia areata and other autoimmune diseases mediated by cytotoxic T cells, in contrast to the alleviation of myasthenia gravis symptoms which is known to be autoantibody-mediated.

## 2. Case Presentation

A 58-year-old male presented to the family medicine clinic complaining of slurred speech. The patient is a known diabetic and has a history of nonalcoholic fatty liver disease (NAFLD). He was referred to neurology, where a complete examination was done including a full body MRI as there was suspicion of stroke and malignancy. The findings were not remarkable. A differential of myasthenia gravis was made and the patient was given pyridostigmine with instructions to return after three days to see if symptoms improved. A diagnosis of myasthenia gravis was further confirmed via a positive serology test for antibodies and a nerve conduction study (NCS). His laboratory findings are summarized in Table 1.

**Figure 1:**
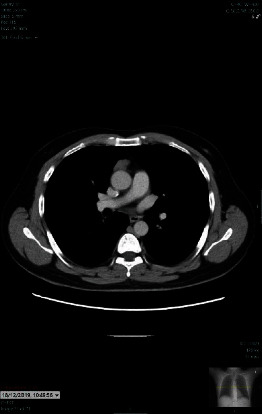
Lobulated soft tissue mass in the anterior mediastinum measuring 1.6 cm × 2.9 cm suggestive of thymoma. No fat components or calcifications. No evidence of pleural metastasis.

A CT with contrast was performed which yielded an enlarged thymus gland and the diagnosis of thymoma-associated myasthenia gravis was made (Figure 1). The patient underwent a thymectomy via thoracotomy in February 2020, and subsequent CTs showed no recurrence or signs of metastasis. Following the thymectomy, histopathological analysis provided further insight into the nature of the thymic lesion. The specimen marked “Thymus Gland” turned out to contain only benign fibrofatty tissue. A separate sample, labeled “Pericardial Fat” by the surgeon, was confirmed as Thymoma B2, measuring 3 cm × 2 cm × 2 cm and demonstrated capsular invasion. Tumor extension into adjacent mediastinal fat reached the inked surgical resection margins, as detailed in Block 2 of the pathology report. These findings suggested microscopic transcapsular invasion, corresponding to a modified Masaoka stage IIa, without evidence of regional lymph node involvement, confirmed by the histological absence of metastasis in all four examined mediastinal lymph nodes. He was also prescribed Imuran (azathioprine) and intravenous immunoglobulin (IVIG). Initially responsive to IVIG, the patient subsequently developed a severe anaphylactic reaction during the last infusion, characterized by acute shortness of breath and extensive skin rash, necessitating emergency intervention and discontinuation of IVIG. This complication significantly altered the course of treatment, leading to the cessation of IVIG and the necessity to adjust his therapeutic approach.

**Figure 2:**
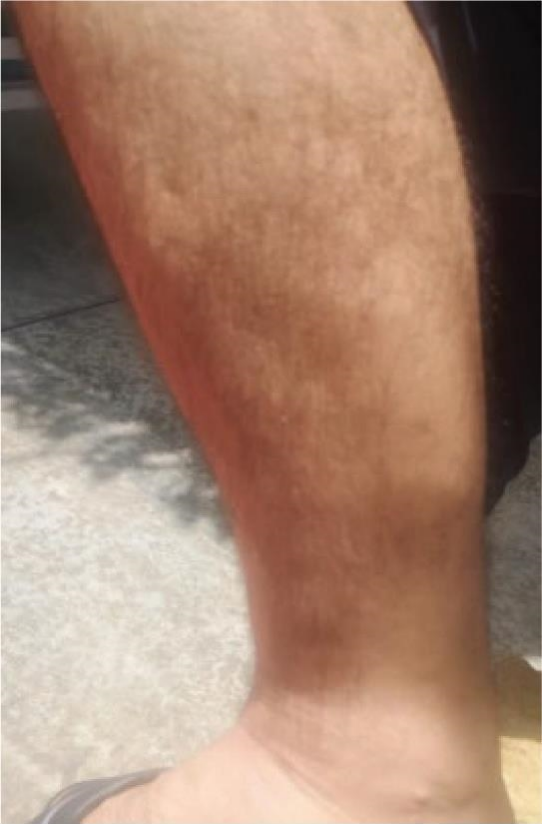
Alopecia areata on the lateral shin after cortisone taper and tacrolimus treatment.

Consequently, the patient was started on a medium dose of cortisone 25 mg, which was then increased to 50 mg as symptoms did not improve. During the cortisone tapering, the patient was maintained on fixed doses of rituximab and tacrolimus. Notably, the pyridostigmine dose was adjusted from 60 mg four times daily (QiD) to 180 mg three times daily (TID). Concurrently, the patient reported progressive hair loss localized to the lateral aspects of both shins. Soon after, in July 2022, he developed further difficulties in chewing/eating, swallowing, speaking, and generalized body weakness, particularly in his proximal muscles. He was admitted to the hospital and was started on six sessions of plasma exchange therapy (PLEX). Rituximab and tacrolimus were added to his prescription. Symptoms improved but returned after two weeks, albeit less severe than on admission. During the time of the high cortisone dose, the patient noticed progressive hair loss on the lateral aspects of both shins (Figure 2). A concurrent diagnosis of alopecia areata was made. He was prescribed tacrolimus ointment.

Over the same period, he experienced weight gain and uncontrolled blood glucose levels and so it was decided to taper off the cortisone. Once the dose was tapered to 10 mg, the patient reported marked improvement in symptoms. His alopecia had also improved significantly and was almost nonexistent once the cortisone was stopped completely. On follow-up, the patient has not had any exacerbations since, but still suffers from slurred speech. In summary, our patient has severe refractory myasthenia gravis with concurrent alopecia areata. He was found resistant to most of the classic medications but surprisingly seemed to improve during the cortisone taper.

**Figure 3:**
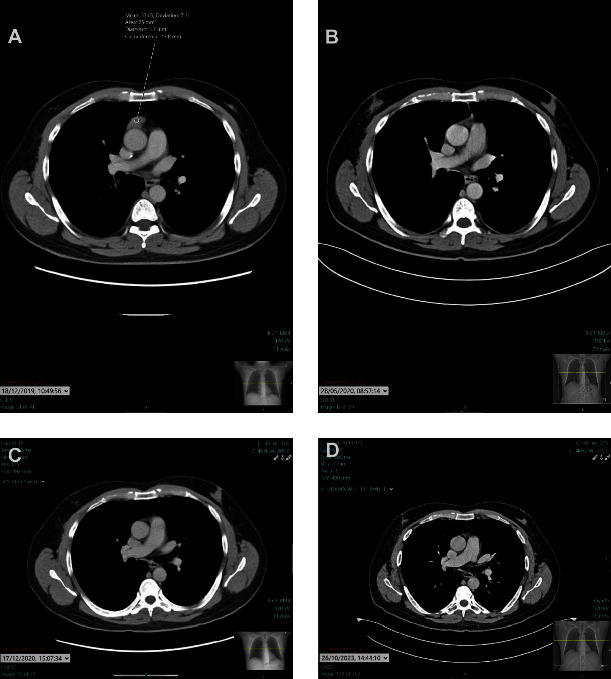
Serial axial CT images of the chest with contrast presented in reverse chronological order, illustrating the patient's progression from the initial diagnosis to postoperative follow-up after thymectomy for myasthenia gravis associated with thymoma. Image A, dated 18/12/2019, shows the prethymectomy appearance of the thymoma. Image B, from 28/05/2020, shows the mediastinal status shortly after thymectomy. Image C, dated 17/12/2020, and Image D, from 26/10/2023, demonstrate the absence of recurrent disease and the continued postoperative integrity of the mediastinal structures, respectively. This composite figure provides a comprehensive overview of the surgical outcome and the successful absence of recurrence over the course of follow-up.

Following the thymectomy, serial CT scans confirmed no recurrence of the thymoma or signs of metastasis, indicating complete surgical excision (Figure 3).

## 3. Discussion

Myasthenia gravis is a common autoimmune disorder targeting the NMJ. The incidence of myasthenia gravis is rather low, with 10–20 new cases per million years [18]. The disease has a bimodal distribution, peaking in the second to third decade in the case of women and the fourth to eighth decade in the case of men [19]. It is classified as a class two hypersensitivity reaction, regulated by IgG autoantibodies against the AChR. This causes a defect in the propagation of action potential (AP) to the postsynaptic AChR, thus preventing the muscles from depolarization. A typical presentation of this disease is variable and fatigable skeletal muscular weakness, mostly affecting the extraocular muscles and, to a lesser extent, the muscles of mastication; therefore, dysphagia is a commonly reported symptom in older males.

An autoimmune condition known as alopecia areata is produced by autoimmune T lymphocytes that attack the tissue of the hair follicles [12]. The patient's quality of life is reduced by alopecia areata, which is defined by CD8-positive lymphocyte infiltration in the hair follicles. Alopecia areata is one of the nonmotor signs of myasthenia gravis-associated thymoma, according to Suzuki, Utsugisawa, and Suzuki [17], yet it may go unnoticed for several reasons.

Myasthenia gravis has been associated with other autoimmune disorders like alopecia areata. Japan, America, and Europe all have different incidences of myasthenia gravis and alopecia areata. 10.2% of all myasthenia gravis patients exhibit alopecia areata, according to Tanaka et al. In contrast, Palmisani et al. [20] reported only two occurrences of alopecia areata among 555 cases of myasthenia gravis patients, while Muller and Winkelmann [21] discovered only two cases of myasthenia gravis linked with thymoma among 736 cases of alopecia areata [20–22]. However, newer studies such as Suzuki et al. shows that an estimated 12% of myasthenia gravis-associated thymoma patients have been reported to have alopecia areata, and the illness is unrelated to the amount of myasthenia gravis activity [23].

In addition to being related to alopecia areata, myasthenia gravis is also associated with thymoma. In around 3% of the cases, thymoma is related to myasthenia graves [24]. Although thymoma and myasthenia gravis are often linked, alopecia areata-complicated thymomas are extremely uncommon. Most of these instances of thymoma with alopecia areata are worsened by myasthenia gravis, which is defined by the infiltration of CD8-positive lymphocytes in the hair follicles [25].

There have been various treatments identified for alopecia areata. It is believed that systemic glucocorticoids are beneficial for rapidly progressing or extensive cases of alopecia areata. According to Kamada, Hatamochi, and Shinkai [12], there have been reports of five cases where thymectomy was performed for thymoma associated with alopecia areata and/or myasthenia gravis. Out of these cases, systemic prednisolone was effective in three cases, both systemic prednisolone and thymectomy were effective in one case, and thymectomy alone was effective in one case where the symptoms of myasthenia gravis and the scalp lesions were connected. In addition, Kamada, Hatamochi, and Shinkai [12] reported a case where alopecia showed significant improvement after thymectomy and steroid medication in a patient with myasthenia gravis-associated thymoma. While the adjustment in pyridostigmine dosage was aimed at better managing myasthenia gravis symptoms, its impact on alopecia areata remains speculative and underexplored in the literature. This highlights a potential area for future clinical research, exploring how NMJ therapies might inadvertently influence autoimmune dermatological manifestations.

One possible explanation for the improvement of the symptoms after cortisone tapering is that corticosteroids have immunosuppressive and anti-inflammatory effects, which can modulate the autoimmune response in myasthenia gravis. Corticosteroids may reduce the production of autoantibodies against AChR, decrease the complement-mediated damage of the NMJ, and inhibit the activation of T cells and B cells [26]. Furthermore, corticosteroids may also have a beneficial effect on alopecia areata, as they can suppress the inflammatory infiltrate around the hair follicles and restore the normal hair cycle [25]. However, corticosteroid therapy is associated with significant side effects, such as osteoporosis, diabetes, hypertension, and infection, and should be used with caution and under close monitoring [26].

In this report, we described a case of uncontrolled myasthenia gravis, that was associated with thymoma and alopecia areata. Our patient presented with symptoms of alopecia over the shin after a diagnosis of uncontrolled myasthenia gravis associated thymoma, following a thymectomy. In contrast to the literature stated above, in our patient, the symptoms of alopecia worsened after the thymectomy and steroid course; he did not develop any autoimmune diseases suggested by previous literature. The literature regarding thymectomy and its role in the treatment of myasthenia gravis-associated thymoma is still unclear and further research needs to be done to determine the pathogenesis and treatment.

## 4. Conclusion

The elderly are vulnerable to misdiagnosis, which might lead to more difficulties. To summarize, this case demonstrates that myasthenia should be considered in older individuals who report muscle weakness despite the absence of conventional symptoms. It is critical to detect myasthenia in its early stages and treat it accordingly. The observed improvements following a cortisone taper suggest its potential therapeutic role in managing such complex cases. The co-occurrence of myasthenia gravis and alopecia areata highlights a possible shared immunological basis, warranting further research. This case highlights the necessity of vigilant monitoring for autoimmune manifestations postthymectomy, advocating for a personalized approach to managing complex immune responses in similar clinical scenarios. This case emphasizes the importance of personalized medicine and the need for continuous exploration of novel treatment approaches in autoimmune disorders.

## Figures and Tables

**Table 1:** Laboratory values of the patient.

Test	Result	Comment
WBC	11.57·10^9^	High
Platelets	144·19^9^	Low
Albumin	38 g/L	Low
Protein	58.8 g/L	Low
Neutrophil (auto)	76.6%	High
Neutrophil absolute (auto)	8.86·10^9^/L	High
Lymphocyte (auto)	13.2%	Low
Lymphocyte absolute (auto)	1.53·10^9^/L	Normal
Monocyte (auto)	9.7%	Normal
Monocyte absolute (auto)	1.12·10^9^/L	High
Eosinophil (auto)	0.2%	Low
Eosinophil absolute (auto)	0.02·10^9^/L	Low
Thyroid-stimulating hormone (TSH)	1.560 mU/L	Normal
Free thyroxin (FT4)	14.8 pmol/L	Normal
C-reactive protein (CRP)	4.9 mg/L	High risk
Creatine phosphokinase (CPK)	Not done	—
Acetylcholine receptor IGg antibodies	3.61 nmol/L	High
Antimuscarinic specific kinase abs MUSK	<0.01 nmol/L	Negative

## Data Availability

The data used to support the findings of this study are available from the corresponding author upon request.
